# Procedural Metacognition and False Belief Understanding in 3- to 5-Year-Old Children

**DOI:** 10.1371/journal.pone.0141321

**Published:** 2015-10-30

**Authors:** Stéphane Bernard, Joëlle Proust, Fabrice Clément

**Affiliations:** 1 Cognitive Science Centre, University of Neuchâtel, Neuchâtel, Switzerland; 2 Institut Jean Nicod, ENS, Paris, France; Max Planck Institute for Human Cognitive and Brain Sciences, GERMANY

## Abstract

Some studies, so far limited in number, suggest the existence of procedural metacognition in young children, that is, the practical capacity to monitor and control one’s own cognitive activity in a given task. The link between procedural metacognition and false belief understanding is currently under theoretical discussion. If data with primates seem to indicate that procedural metacognition and false belief understanding are not related, no study in developmental psychology has investigated this relation in young children. The present paper aims, first, to supplement the findings concerning young children’s abilities to monitor and control their uncertainty (procedural metacognition) and, second, to explore the relation between procedural metacognition and false belief understanding. To examine this, 82 3- to 5-year-old children were presented with an opt-out task and with 3 false belief tasks. Results show that children can rely on procedural metacognition to evaluate their perceptual access to information, and that success in false belief tasks does not seem related to success in the task we used to evaluate procedural metacognition. These results are coherent with a procedural view of metacognition, and are discussed in the light of recent data from primatology and developmental psychology.

## Introduction

In developmental psychology, exploring metacognition, that is, the ability to evaluate one’s own cognitive activity and to act accordingly, classically relies on explicit epistemic self-evaluations made by school-aged children (for a review, see [1]). Reliable metacognitive awareness has long been considered to be highly dependent on children’s understanding of their having mental states [2][3]. The assumption was that one cannot properly monitor and control one’s own cognitive activity unless one is able to reflectively represent that one has first-order mental states, that is, unless one can metarepresent them as perceptions, memories, judgments, and so forth with specific contents [4][5]. In this view, a definition of metacognition as “thinking about thinking” [6] is taken to imply that one possesses the concept of knowledge—applied, for example—to one’s perception or one’s memory [7][8]. Given the additional assumption that a metacognitive ability is best expressed in a verbal report about what one thinks, perceives, and so forth, very little research has focused on the availability of non-verbal metacognitive processes in younger children [9].

Alternative paradigms, however, have more recently been used to test implicit abilities of young children to evaluate and control their own cognitive activity, that is, procedural metacognition. These paradigms, first used with non-human animals, build on the assumption that, while concept-based metacognitive capacities appear in children at around 6 to 8 years of age (e.g., [1]), a form of experience-based, or procedural, metacognition allows younger children to assess what they can perceive or remember in an activity-dependent, rather than concept-based way.

In one of these experimental paradigms, participants are requested to indicate whether or not they are willing to answer a given question or perform a given task (e.g., categorize a perceived stimulus, retrieve a matching icon from memory). In a procedural view of metacognition, participants in such opt-out tasks can reliably use a non-conceptual type of information in making such a decision: the feeling of ease (or difficulty) experienced when trying to perceive a target stimulus (or remember an item, etc.), independently of any self-reflection or interpretation [10][11][12].

In animal studies, opt-out paradigms, along with other paradigms (such as wagering and information seeking), have demonstrated that uncertainty is reliably monitored in monkeys and apes to contextually control perceptual discrimination and memory retrieval ([13][14]; see [15] for a review). Although some studies that aimed at testing procedural metacognition in dolphins, rodents, and birds are still being debated for their methodology [7][16][17], stricter experimental paradigms applied to rhesus monkeys have offered strong evidence in favor of the existence of procedural metaperception and metamemory in some primate species: They yield response patterns similar to those of humans when evaluating their own uncertainty with respect to a first-order cognitive task, for example, a task of perceptual discrimination where cognitive performance needs to be evaluated predictively (in order to choose whether to perform a task or skip it), or retrospectively (for wagering on the correctness of a cognitive outcome) [18][19]. In contrast with a concept-based approach to metacognition, however, these animals cannot be credited with a capacity to reflectively represent that they have first-order mental states. They are, rather, consistently found to fail to attribute false beliefs to conspecifics, which presumably implies that they are unable to form metarepresentations of epistemic states in themselves as well as in others [20][21].

In developmental studies, some authors have investigated uncertainty monitoring and control, that is, procedural metacognition, using a memory task [22][23], a visual discrimination task [24][25], or an information-seeking task [26]. For instance, 3-, 4-, and 5-year-olds demonstrated uncertainty monitoring by reporting higher subjective uncertainty for inaccurate versus accurate responses on a perceptual identification task [24]. This study, however, exclusively targeted uncertainty monitoring. In another study, Lyons and Ghetti [25] tested both uncertainty monitoring and control, using again a visual discrimination task. In a free-choice condition, 3- to 5-year-old children had to decide whether or not to select one drawing from two degraded drawings as depicting a target item (e.g., a bed), and to rate their confidence in their answer. To familiarize them with the explicit judgment of confidence scale, children received a practical metacognitive training involving positive or negative feedback. Subsequently, practice trials with feedback were also completed on the accuracy of their selection, with no confidence judgment collected. During these trainings, children were presented with items similar to those used in the test phase. The results of the free-choice condition showed that 3- to 5-year-old children, without significant age group differences, selectively withhold responses for trials in which they report being uncertain. In other terms, children present both a capacity to monitor their uncertainty and a capacity to select a response strategy on this basis.

While Lyons and Ghetti [25] found a positive relation between uncertainty monitoring and explicit confidence judgments, another study failed to find one. Using a memory task with 3.5-year-old children, Paulus et al. [23] found that explicit confidence judgments did not differ for remembered and non-remembered items. In contrast, assessments of implicit confidence (measured with fixation time and pupil dilations) were affected by the children’s evaluation of their memory: Children (a) looked longer to high-confidence ratings when they correctly remembered the associated item, and (b) had pupil dilations for correctly remembered items, but not for incorrectly remembered items. These last results suggest that implicit confidence assessments may develop earlier than explicit assessments. Nevertheless, it is arguably unclear that, in Lyons and Ghetti [25], the children’s explicit confidence evaluations were prompted by children’s metarepresentations of their own states of knowledge rather than by a procedural type of assessment. In the first case, participants would need to form a second-order belief about their current first-order memorial state in order to evaluate whether the latter is likely to be correct or not. They would thus need to represent their current performance as an attempt to remember an item. In the second case, however, no such metabelief is needed to mediate a metacognitive evaluation. Participants merely have to engage in a task and make predictions on its likely correctness on the basis of activity-dependent cues (such as noetic feelings). In this alternative view, the informational cues being used by participants are not conceptual but experience-based. One way to shed light on what is at stake in Lyons and Ghetti’s study can be to investigate the relation between procedural metacognition and the ability to reflectively represent first-order mental states. This kind of ability can be tested thanks to false belief tasks. So far, the relation between procedural metacognition and false belief understanding has not been made in a developmental perspective with preschoolers.

Whether metacognition is procedural or, rather, linked to the ability to reflectively represent first-order mental states, it presupposes that participants assess their ability in performing a cognitive task, which may be perceptual, memorial or other. Balcomb and Gerken [22] adapted a paradigm initially used with rhesus monkeys (Shields, 1999; in [27]) to explore procedural metacognition in 3.5-year-olds. Children first had to learn pairs of associated items. In the subsequent metamemory task, the children, presented with the first of the two previously associated items, had to decide either to complete the task (and thus indicate the second item in the pair) or to opt out. In the second test phase, the same children were subjected to a forced-choice recognition task (no opting out allowed). They had to recognize whether a presented object had been paired with a particular item.

Results showed that accuracy for accepted items (evaluated in the metamemory task) was significantly higher than accuracy for skipped items (evaluated in the recognition task). Balcomb and Gerken’s study thus showed that 3.5-year-olds, who have difficulty reporting verbally what they do or do not know [1][28][29], can nevertheless access their knowledge in a procedural way (i.e., non-verbally) when they have to decide whether or not to complete a particular memory trial.

The present experiment first aims to extend Balcomb and Gerken’s findings by investigating young children’s ability to monitor and control their uncertainty (procedural metacognition) by using a new visual discrimination task: Is the children’s accuracy significantly higher for accepted than for rejected items when performing a forced recognition task? Granting that an opt-out task should be based on feelings of uncertainty experienced while performing the task rather than on a concept-based assessment of what one manages to discriminate, our task should elicit a procedural form of metacognition. In contrast with Lyons and Ghetti’s study [25], no practical metacognitive trainings involving positive or negative feedback were presented to our participants to avoid any influence of training on the children’s subsequent performance during the test phase.

A second aim of our study is to explore whether procedural metacognition in our opt-out task is linked to the ability to reflectively represent first-order mental states. To do this, participants are presented both with the visual discrimination task and with three false belief tasks. Although a false belief task generally consists in assessing others’ epistemic states, it has also been used to assess one’s own epistemic states because self- and other-directed metarepresentations have similar conceptual requirements.

Given, on the one hand, that little is known about how procedural metacognition develops and, on the other hand, that children’s false belief understanding critically improves during the preschool period, the present experiment involves 3- to 5-year-old children.

## Ethics Statement

We conducted our research after approval and under the supervision of the University of Neuchâtel. All participants were treated according to the Declaration of Helsinki. The University of Neuchâtel Ethical Review Committee ethically approved the present experiment and the consent procedures. For each child participant, we obtained written informed consent from parents for the participation of their child, and child participants verbally agreed to participate before the beginning of the study. For each adult participant, we obtained written informed consent before participation.

## Method

### Participants

This experiment involved 82 children: 27 3-year-olds (11 girls, *M*age = 42.92 months, *SD* = 3.51, range 36–47 months), 26 4-year-olds (11 girls, *M*age = 52.76 months, *SD* = 3.76, range 48–59 months), and 29 5-year-olds (16 girls, *M*age = 66.72 months, *SD* = 3.63, range 60–71 months) from two schools in a French-speaking city. Most children came from middle- and upper-middle-class families. Each child was seen individually in a quiet room by a single experimenter for about 10 minutes.

### Materials and procedure

#### Visual discrimination task: Opt-out paradigm

To build the stimuli, we selected 15 pictures from a children’s book. We chose these pictures because they depicted objects well-known to 3- to 5-year-old children. These pictures were modified (software Photofiltre) to become mosaic pictures (by pixel modification) with several degrees of discriminability. Thanks to this software, we obtained four levels of difficulty for the same item. Pictures were modified with a tile size of 6 to obtain the pictures of Level 1 (*N* = 15), a tile size of 12 for Level 2 (*N* = 15), a tile size of 18 for Level 3 (*N* = 15) and a tile size of 24 for Level 4 (*N* = 15). Once we constructed these stimuli (*N* = 60), a second step was to validate them in an adult population with a pre-test.

Thirty adults were recruited for this pre-test (15 women, *M*age = 32.01 years, *SD* = 5.79 years, age range 23–51 years). Before presenting the 60 pictures, the experimenter explained to the adults that they would see some pictures and asked them to report what each picture depicted, in their opinion, on a response sheet. Pictures were presented on a computer screen and a black screen appeared between each picture to let the adults write their response. The set of 15 more difficult items (level 4) were first presented; they were followed by the set of less difficult items of level 3, then of level 2, and finally of level 1. At each level, the 15 items were presented in a counterbalanced order.

A repeated measures ANOVA with one within-subjects factor, difficulty level (4, 3, 2, 1), and one between-subjects factor, gender (female, male), revealed only a main effect of difficulty level: *F*(3,26) = 344.96, *p* < .001, *η* ^*2*^ = .92. The performance at Level 1 (99.1% of correct identification, *M* = 14.87, *SD* = 0.43) was significantly better than performances at Level 2 (72.5%, *M* = 10.87, *SD* = 1.79, *p* < .001) (Note that all comparisons after an ANOVA were calculated according to the Bonferroni procedure). The performance at Level 2 was significantly better than those at Level 3 (46.8%, *M* = 7.03, *SD* = 2.18, *p* < .001). Finally, the performance at Level 3 was significantly better than that at Level 4 (29.5%, *M* = 4.43, *SD* = 1.86, *p* < .001). Given these results, we created test stimuli for children by selecting three representative items for each level of difficulty. One can argue that the procedure used to evaluate the 60 pictures may have led to an effect of presentation order. For each picture from the children’s book (*N* = 15), adults first viewed the set of 15 more difficult items (level 4), then the set of 15 less difficult items (Level 3) and so on until the level 1. Thus adults could have better identified the items at an easier level (e.g., level 2) than at more difficult ones (e.g., level 4), partly because they had already encountered the items previously. The percentage of correct identification for each level has thus to be considered with this limitation in mind. Nevertheless, the procedure used allowed the selection of representative items for each level of difficulty.

For each level, three different items were chosen (e.g., cat, flower, and car at Level 4). For each item selected, there was no significant difference between the general mean for the level selected (Level 4 for instance) and the mean of the item selected at this level (means obtained with adults). For instance, the mean for cat at Level 4 was .23, and the general mean of Level 4 was .29; accordingly, we held that cat was a representative item for Level 4. Twelve items were selected in this way as test items for the children (three items per level): chicken, plane and tree for Level 1; bird, bed and fire for Level 2; rabbit, house and grapes for Level 3; cat, car and flower for Level 4. The same 12 items were used for each age group.

Tests with both adults and children were conducted with the same presentation size (602 × 620 px). The visual discrimination task was divided into three sub-tasks: an opt-out task, a recognition task, and an identification task.

In the opt-out task, the general framework was the following. For each item (*N* = 12), children were shown a picture and invited to either press a green button if they recognized the picture or a red button if not (to skip the trial). The experimenter said to the children, “If you know what it is, press the green button; if you do not know what it is, press the red button” (this instruction was only used during the two warm-ups). If they pressed the green button, the experimenter asked the children to name the stimulus. If they pressed the red button, the next stimulus appeared on the screen. The goal of this task was to determine whether children can act strategically on the basis of uncertainty. This opt-out task consisted of two warm-ups and then the task per se.

The warm-ups were very helpful because younger children were prone to respond immediately when they knew what the image depicted. After the warm-ups, however, children generally succeeded in executing the procedure as they were instructed to.

In the first warm-up, children were shown four pictures without modifications in a counterbalanced order in order to familiarize them with the procedure and with the use of each button. There were two pictures of familiar objects (a pear and a dog) for the green button use: Since children at these ages generally know the name of these two objects, they pressed the green button, after which they were asked to give their response aloud. The stimuli used in this first warm-up also included two pictures of unknown objects to train the children in using the red button: Given that children were not able to say what the unknown object was, they pressed the red button, after which they were allowed to see the next item.

In the second warm-up, children were shown four pictures (one for each level) in a counterbalanced order in order to familiarize them with the modified pictures as well as to continue to familiarize them with the button use. After these two warm-ups, the test items of the opt-out task were presented to the children in a counterbalanced order. No positive or negative feedback was provided to the children during the two warm-ups. For each item of these two warm-ups, however, the experiment repeated the instruction presented above: “If you know what it is, press the green button; if you do not know what it is, press the red button.” No instruction or feedback was provided for the test items.

After children saw all the items of the opt-out task, a black screen appeared and the experimenter explained that they were going to see the same pictures (*N* = 12). This time, however, they just had to say aloud what, according to them, the images depicted, without using the buttons. This recognition task was used to assess the accuracy of the responses to items respectively accepted or rejected during the opt-out task. The two former tasks were created with the software program E-Prime.

This program was used (a) to record which button (green or red) was pressed by children during the opt-out task, (b) to collect data concerning whether children responded correctly after having pressed the green button in the opt-out task (we clicked on the right or left button of a mouse to record whether children gave a correct response [left] or an incorrect response [right]), (c) and to collect data about whether children responded correctly in the recognition task (same mouse procedure).

When children failed to name an object in the opt-out task and in the recognition task, the same, non-modified picture of this object was presented to them on a plastic card to control for their capacity to name it. This identification task was used to ensure that failures in the opt-out task and/or the recognition task were not due to lacks in vocabulary. Only the children who had either correctly labeled all the pictures in this task, or who did not have to pass this task (because they had correctly named all the items in the two previous tasks) were included in the analyses.

#### False belief tasks

In this experiment, children were also presented with three false belief tasks. The first task was an unexpected transfer task adapted from Wimmer and Perner [30]. In this task, a story character put an object in one place and then left. Another character then moved the object to another location; after this, the first character came back. The child was asked a test question (an “other’s belief” question): “Where will [story character] look for her [object]?” Two control questions were asked to check whether the child remembered both the original location (“Where was [object] in the beginning?”) and the present one (“Where is the [object] now?”). These control questions had to be answered correctly for credit to be given to the participant for the test questions. The test question was allotted a binary score (1/0). Children obtained 1 point when they predicted that the character will search for the object according to her false belief (i.e., indicating the first location of the object).

The second task was an unexpected content task adapted from Perner, Leekam and Wimmer [31]. In this task, the child had to identify the expected content of a familiar box. After having answered what the expected content was (e.g., Smarties with a Smarties box), the child could open the box and look at the unexpected content inside it. Then the box was closed and the child was asked two test questions: a “self’s belief” question: “Before opening the box, what did you think was inside?”, and an “other’s belief” question about a friend who had not looked inside the box: “[name of the friend] has not looked inside the box. What will he/she think is inside the box before opening it?” A control question was asked in order to check whether the child remembered the actual content of the box (“What is there in the box?”). As in the first task, this control question had to be answered correctly before credit could be given to the participant for the test questions. Each test question (*N* = 2) was allotted a binary score (1/0). For each test question, children obtained 1 point when they referred to the expected content of the box (e.g., Smarties).

The third task was also an unexpected task, adapted from Jenkins and Astington [32]. In this task, a child was shown a picture book in which a partial view of what seemed to be a rabbit’s tail was in fact a lady’s hair bun (unexpected picture). When the page was turned, however, he could get a full view of the picture. The child was asked the following test questions: a “self’s belief” question, “Before I turned the page, what did you think it was?”, and an “other’s belief” question about a friend, “[name of the friend] has not seen this image. Before I turn the page, what will he/she think it is?” Each test question (*N* = 2) was allotted a binary score (1/0). For each test question, children obtained 1 point when they referred to the expected picture (the rabbit’s tail). For each false belief task, the number of correct responses was divided by the number of test questions. For these three tasks, the child could therefore obtain a maximum raw score of 3.

This false belief evaluation was made in order to test the relation between children’s procedural metacognition and their false belief understanding.

## Results

Our first aim, with respect to the data analysis, was to compare two different results: those related to the accepted items in the opt-out task and those related to the rejected items in the recognition task. Our hypothesis was that accuracy for accepted items should be significantly higher than accuracy for rejected items, that is, that children could reliably assess their discriminative ability in a given trial.

Our second aim was to test the relation between procedural metacognition and false belief understanding. To do this, we created a differential score tallying accuracy for accepted items minus accuracy for rejected items. This score should express children’s performance level in procedural metacognition.

### Accepted versus rejected items

**Fig 1:**
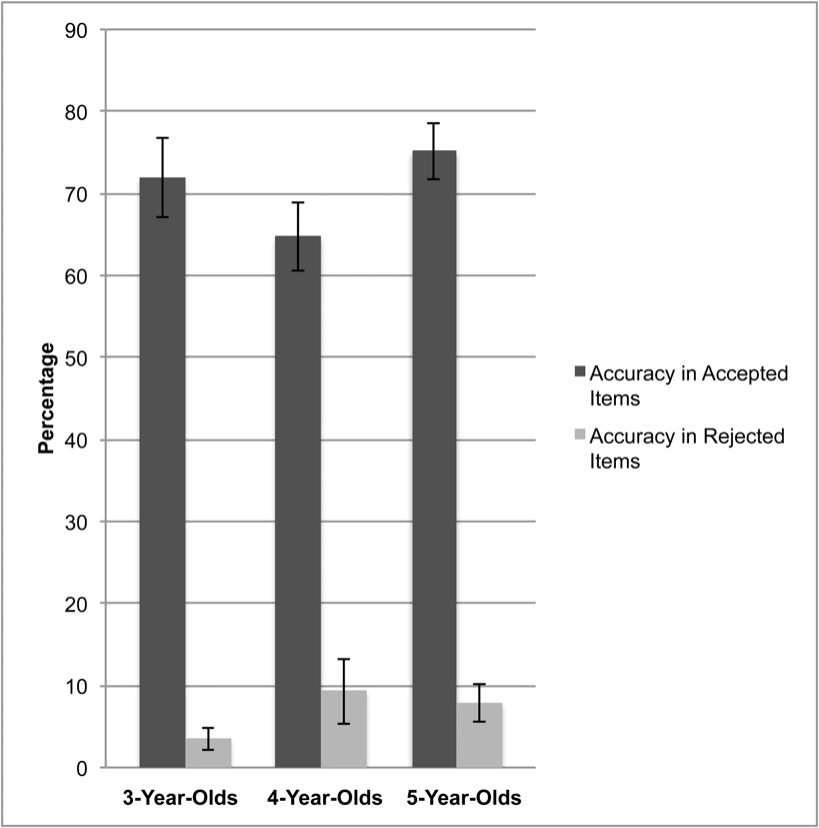
Percentage of accuracy for accepted and rejected items in each age group.

Only one child (a 3-year-old) failed to name two pictures in the identification task and was thus removed from the analyses. Moreover, one 3-year-old and one 5-year-old accepted all trials; their data were not included in the analyses because they did not reject any item. No child declined all trials (S1 File). In sum, 79 children accepted and declined at least one trial. With respect to the percentage of accepted items in the opt-out task, an ANOVA revealed no significant effects of age group, *F*(2,73) = .84, *p* = .436, *η* ^*2*^ = .023, or gender, *F*(1,73) = .077, *p* = .782, *η* ^*2*^ = .001. The age group X gender interaction was not significant: *F*(2,73) = .153, *p* = .859, *η* ^*2*^ = .004. With respect to accuracy for accepted items (Fig 1), another ANOVA also revealed no significant effects of age group, *F*(2,73) = 1.29, *p* = .28, *η* ^*2*^ = .034, or gender, *F*(1,73) = .13, *p* = .718, *η* ^*2*^ = .002. The age group X gender interaction was not significant: *F*(2,73) = 1.88, *p* = .160, *η* ^*2*^ = .049. For all children, the choices of the green button (and hence the choices to label the picture) were at 53.5%. The responses for these accepted items were accurate to 70.6%.

With respect to accuracy for rejected items (Fig 1), an ANOVA revealed no significant effects of age group, *F*(2,73) = 1.21, *p* = .305, *η* ^*2*^ = .032, or gender, *F*(1,73) = 3.02, *p* = .086, *η* ^*2*^ = .040. The age group X gender interaction was not significant: *F*(2,73) = 1.25, *p* = .293, *η* ^*2*^ = .033. For all children, the responses concerning these rejected items in the recognition task were accurate to 6.9%.

We conducted the next analysis to determine whether children were more accurate in their perceptual discriminations for accepted items than for rejected items, that is, whether they were able to reliably monitor and control their perceptual uncertainty. An ANOVA revealed no significant main effects of age group, *F*(2,73) = .66, *p* = .518, *η* ^*2*^ = .018, or gender, *F*(1,73) = 1.54, *p* = .218, *η* ^*2*^ = .021, and a significant main effect with respect to accepted versus rejected items, *F*(1,73) = 481.45, *p* < .001, *η* ^*2*^ = .87. The age group X accepted/rejected items interaction was not significant: *F*(2,73) = 1.92, *p* = .153, *η* ^*2*^ = .05; the gender X accepted/rejected items interaction was not significant: *F*(1,73) = .45, *p* = .503, *η* ^*2*^ = .006; the age group X gender X accepted/rejected items interaction was not significant: *F*(2,73) = 2.47, *p* = .092, *η* ^*2*^ = .063. The performance with the accepted items (*M* = .71, *SD* = .21) was significantly better than that for the rejected items (*M* = .07, *SD* = .14, *p* < .001). As predicted by the descriptive results (Fig 1), children were significantly more accurate on accepted items.

### Procedural metacognition and false belief understanding

Mean performance in procedural metacognition (expressed by the differential score computing accuracy in accepted items minus accuracy in rejected items) was at 68.4% for the 3-year-olds, 55.6% for the 4-year-olds, and 67.3% for the 5-year-olds.

With regard to the three false belief tasks, 11 children were not able to respond correctly to the control questions: five 3-year-olds, five 4-year-olds and one 5-year-old. They were thus removed from the following analyses. The mean performance was at 35% correct answers for the 3-year-olds, 56.3% for the 4-year-olds and 75.3% for the 5-year-olds. An ANOVA revealed a significant effect of age group, *F*(2,62) = 10.31, *p* < .001, *η* ^*2*^ = .25, and no significant effect of gender, *F*(1,62) = .17, *p* = .67, *η* ^*2*^ = .003. The age group X gender interaction was not significant: *F*(2,62) = 2.37, *p* = .10, *η* ^*2*^ = .071. The 3-year-olds chose the correct answer significantly less often (*M* = 1.05, *SD* = .97) than the 5-year-olds (*M* = 2.26, *SD* = .66, *p* < .001) and the 4-year-olds (*M* = 1.69, *SD* = 1.15, *p* = .044) did. The 4-year-olds’ performance was not significantly different from the 5-year-olds’ performance (*p* = .178). Three-year-olds answered below chance in their choices of the correct answer: *t*(19) = -2.07, *p* = .052, *d* = -.94. Four-year-olds responded at chance, *t*(20) = .75, *p* = .46, *d* = .33, and 5-year-olds answered above chance, *t*(26) = 6.01, *p* < .001, *d* = 2.36).

Given that this false belief evaluation was conducted to test the relation between procedural metacognition and false belief understanding, the next step in our analysis aims to examine (a) whether children who succeeded in the false belief tasks had better performance in procedural metacognition (i.e., in the differential score) than those who failed in the false belief tasks, (b) the correlations between the differential score and false belief scores, and (c) the relation between these two variables within a multiple regression analysis framework.

The percentage of children who succeeded in the false belief evaluation (i.e., having more than 1.5 points on the maximum raw score of 3) was at 25% for the 3-year-olds, 57.1% for the 4-year-olds, and 85.2% for the 5-year-olds. The performance of children who succeeded in the false belief evaluation and those who did not (i.e., having less than 1.5 points) was not significantly different for the differential scores, for all children (*M*success = .63, *M*failure = .63, *t*(66) = .085, *p* = .93, *d* = .02), and for each age group (3-year-olds: *M*success = .64, *M*failure = .72, *t*(18) = .62, *p* = .54, *d* = .29; 4-year-olds: *M*success = .53, *M*failure = .49, *t*(19) = -.26, *p* = .79, *d* = -.12; 5-year-olds: *M*success = .68, *M*failure = .63, *t*(25) = -.47, *p* = .64, *d* = -.19).

Regarding the correlation, there was no significant correlation between the differential scores and the false belief scores, for all children (*r*(68) = .08, *p* = .52) and for each age group (3-year-olds: *r*(20) = .07, *p* = .77; 4-year-olds: *r*(21) = .16, *p* = .48; 5-year-olds: *r*(27) = .06, *p* = .77).

Finally, we examined whether false belief scores and chronological age predicted differential scores. Results of a multiple regression, with these two variables entered simultaneously, showed that the model was not significant: *F* (2, 65) = .22, *p* = .806, adjusted R2 = -.02.

## Discussion

The first aim of the present experiment was to extend the to-date limited findings about procedural metacognition in 3- to 5-year-old children. We investigated children’s abilities to monitor and control their uncertainty by using an opt-out task. Results indicate that preschoolers can rely on procedural metacognition to evaluate their perceptual access to information. Children as young as 3 years of age were able to accept the task of naming a stimulus when the informational quality of the stimulus was sufficient and to skip a trial when they were too uncertain. Our results thus offer a metaperceptual complement to Balcomb and Gerken [22]’s experiment on young children’s metamemory, where young children are found to be able to reliably monitor their memory for paired items and to control their decision accordingly (accepting or skipping stimuli).

What, then, is the informational source that children are using to monitor their perceptual uncertainty and to control their responses on this basis? Some researchers claim that these judgments crucially depend on self-reflective awareness [25], which is assumed to depend on the child’s construal of his or her mind “as a representational and interpretive device” (p. [28; 33]), and, more specifically, on a child’s ability to reflectively represent first-order mental states [34].

Our second aim was to explore whether procedural metacognition was linked to the ability to reflectively represent first-order mental states (for relevant debates, see [5][12][35]). To do this, we subjected children to an opt out task and to standard (own/others’) false belief tasks. Results indicate that: (a) Children who succeeded in the false belief evaluation and those who did not have the same level of performance in the opt-out task; (b) no significant correlation was found between children’s abilities to use procedural metacognition and their false belief scores; (c) false belief scores did not predict the performance in the opt-out task. Consequently, the results from our experiment suggest that the ability to reflectively represent first-order mental states, at least when measured with false belief tasks, does not seem to be involved in young children’s success in tasks involving procedural metacognition. These results are coherent with a procedural view of metacognition, in which feelings of uncertainty can guide perceptual decisions independently of any concept-based self-reflection or interpretation [10][11]. These results are coherent with data from primatology. While primates consistently fail to solve false belief tasks [20][21], they have been found able to monitor their first-order perceptual and memorial states ([13][14][15]).

One can object, however, in the light of recent evidence concerning the implicit understanding of belief in infants and toddlers, that our false belief tasks, based as they are on verbal report, do not allow implicit competences in mindreading to be manifested [36][37]. Eighteen-month-olds were found to reliably predict specific action mistakes through false belief attribution to the agent and intervene accordingly [38]. A further step might then be to use implicit tasks in order to elicit forms of implicit understanding of belief in younger children, and to study their correlation with implicit forms of perceptual metacognition. Two caveats about this direction of research, however, are in order. First, it is unclear that the implicit forms of mindreading presumed to appear slightly before the second year of age involve understanding one’s own and others’ cognitive states as mental states. Toddlers might rather succeed in the proposed tasks by relying on predictive behavioral cues or by relying on lower-level associations [12][39][40][41][42]. Second, granting the existence of procedural metacognition in rhesus monkeys ([13][14][15]), early forms of metacognition in humans should perhaps no more depend on the subjects’ intuitive understanding of their own cognitive states as mental states than they do in rhesus monkeys.

In conclusion, our results enrich the to-date limited literature about procedural metacognition in young children (e.g., [22][23][25]). Children’s performances suggest that metacognitive monitoring and control are available in a procedural form when the participants need to evaluate their perceptual abilities in a discrimination task in order to decide what to do. This form of metacognition seems to be unrelated with false belief understanding. Procedural metacognition might precede the development of explicit metacognitive knowledge, and might, in addition, constitute a precondition for it.

Future research about children's procedural metacognition, being still at an early stage, has many open questions to address. First and foremost, new experimental paradigms are needed to elicit existing skills through non-verbal stimuli. Innovative and motivating tasks might be used in order to compare prospective and retrospective evaluation on a same first-order task in a variety of domains, from perception and memory to language understanding and reasoning. Another important research goal will be to investigate whether children's procedural metaperception is influenced by educational and other cultural practices.

## Supporting Information

S1 File All data for the visual discrimination task and the three false belief tasks. (XLSX)
